# Supplementary material for: Magneto-optical Evidence of the Tilting Effect in Coupled Weyl Bands
Source: Nano Lett. 2025 Feb 7;25(7):2858–63. doi: 10.1021/acs.nanolett.4c06072 (PMC11849037; doi:10.1021/acs.nanolett.4c06072)
Supplement: Supplementary file 1 — nl4c06072_si_001.pdf [file nl4c06072_si_001.pdf]

Supporting information: Magneto-optical evidence of tilting effect in coupled Weyl bands

Seongphill Moon,[†,‡] Yuxuan Jiang,[*,¶,§] Jennifer Neu,[†,‡,∥] Theo Siegrist,[†,⊥] Mykhaylo Ozerov,[†] Zhigang Jiang,[*,#] and Dmitry Smirnov[*,†]

†*National High Magnetic Field Laboratory, Tallahassee, Florida 32310, USA*

‡*Department of Physics, Florida State University, Tallahassee, Florida 32310, USA*

¶*School of Physics and Optoelectronic engineering, Anhui University, Hefei, Anhui 230601, China*

§*Center of Free Electron Laser and High Magnetic Field, Anhui University, Hefei, Anhui 230601, China*

∥*Oak Ridge National Laboratory, Oak Ridge, Tennessee 37830, USA*

⊥*Department of Chemical and Biomedical Engineering, FAMU-FSU College of Engineering, Tallahassee, FL, 32310, USA*

#*School of Physics, Georgia Institute of Technology, Atlanta, Georgia 30332, USA*

E-mail: yuxuan.jiang@ahu.edu.cn; zhigang.jiang@physics.gatech.edu; smirnov@magnet.fsu.edu

Sample Growth

Single crystal NbP was grown by chemical vapor transport reaction. Silica tubes with 14 mm inner diameter and 18 mm outer diameter were loaded with Nb foil and P chunks in a stoichiometric ratio such that the total reaction mass was 1 g. $\sim$3.6 mg/cm^3 I_2 crystals were added as transport agent, and tubes were sealed under a vacuum of 10^{-5} mTorr at lengths of $\sim$10 cm. The ampoules were then loaded into a single-zone tube furnace at an angle of $\sim 20°$ against the horizontal for convective flow. The temperature was increased from room temperature to 550 °C at a rate of 5 °C/h, where it was held constant for 24 hours and subsequently heated to the reaction temperature of 950 °C at 2.5 °C/h. Crystal growth proceeded over 3 weeks in a temperature gradient of 950°C : 900°C. Power to the furnace was then turned off, and the ampoules were allowed to cool to room temperature. The structure type of the sample was confirmed using single-crystal X-ray diffraction, and the stoichiometry was confirmed using energy dispersive X-ray spectroscopy employing a Zeiss 1540 XB Crossbeam Scanning Electron Microscope.

Landau Level Calculation

To calculate the Landau level (LL) spectrum of tilted coupled Weyl points in different magnetic field ($\mathbf{B}$) orientations, we first replace the momentum vector $\mathbf{p}$ in Eq. (1) of the main text with $\mathbf{\Pi} = \mathbf{p} + e\mathbf{A}$, where e is the elementary charge, and $\mathbf{A}$ is the vector potential satisfying $\mathbf{B} = \nabla \times \mathbf{A}$. We then rewrite the Hamiltonian H with ladder operators: For $B \parallel k_x$ (or $B \parallel k_w$, where k_w describes the orientation of the coupled Weyl points), we have $\Pi_y + i\Pi_z = (\sqrt{2}\hbar/l_B)a^+$ and $\Pi_y - i\Pi_z = (\sqrt{2}\hbar/l_B)a$, where $\hbar$ is the reduced Planck constant, and $l_B = \sqrt{\hbar/eB}$ is the magnetic length. For $B \parallel k_y$ (or $B \perp k_w$), we have $\Pi_z + i\Pi_x = (\sqrt{2}\hbar/l_B)a^+$ and $\Pi_z - i\Pi_x = (\sqrt{2}\hbar/l_B)a$. Next, we expand the Hamiltonian eigenfunctions with harmonic oscillator wavefunctions up to 100 terms.[1,2] By utilizing the ladder operator relations, we can transform the Hamiltonian into an algebraic matrix and

solve for its eigenvalues. In this case, we can calculate up to the 100th LL.

Magneto-absorption Spectra Calculation

With the LLs and their wavefunctions calculated from the above procedure, we can further compute the magneto-absorption spectra α at frequency ω using Fermi golden rule,[3,4]

$$\alpha(E = \hbar\omega) \propto \sum_{n,m,\mu} \frac{B}{\hbar\omega} |\langle m| v_\mu |n\rangle|^2 \times \frac{f(E_m) - f(E_n)}{\hbar\omega - (E_n - E_m) + i\Gamma},$$

where $v_\mu = \partial H/\partial(p_\mu)$ is the velocity operator, $f(E) = [\exp(\frac{E-E_F}{k_B T}) + 1]^{-1}$ is the Fermi-Dirac distribution, k_B is Boltzmann constant, and E_F is Fermi energy. The matrix element $\langle m| v_\mu |n\rangle$ represents the probability of a transition between the initial $|m\rangle$ and final $|n\rangle$ LLs, with μ denoting the light polarization. We assume that the incident infrared light is equally polarized in the plane perpendicular to the propagation direction. The Fermi-Dirac distribution $f(E_m)$ describes the occupation of the mth LL with energy E_m at liquid helium temperature ($T = 4.2$ K). The parameter Γ represents a phenomenological broadening of the LL transitions, set as a constant ($\Gamma = 1$ meV) in our calculations.

Lastly, we calculate the evolution of the Fermi level in a magnetic field, represented by the red dash lines in Figures 4b and 4d of the main text. Based on the LLs and their dispersions along the magnetic field direction for both $B \parallel k_w$ and $B \perp k_w$, we determine the Fermi level for a given carrier density by accounting for contributions from the LLs in both configurations simultaneously.

Normalized Spectra from Magneto-reflectance Measurements

In Figure 2a of the main text, we present the normalized magneto-reflectance measurement results, $R(B)/R(B = 0\text{T})$, as a function of magnetic field and photon energy. For reference, Figure S1(a) displays the raw spectra used for this plot, while Figure S1(b) is a replot of Figure 2a. Similarly, Figure S1(c) shows the second derivative spectrum, $-d^2R/dE^2$, of each magnetic field, and Figure S1(d) is a replot of Figure 2b from the main text. In Figures S1(a) and S1(c), spectra are vertically offset for clarity. The measurements were taken with a 1 T step size, ranging from 1 T to 17 T.

To extract the symbols (transition energies) in Figure 2b of the main text, we follow this procedure: First, we smooth the raw spectra from Figure 2a using a built-in smoothing method in IgorPro software (binomial algorithm, smoothing factor of 320). Then, we take the negative second derivative of the smoothed data with respect to energy (i.e., $-d^2R/dE^2$). Finally, we identify the peak positions as the corresponding transition energies. These peak positions align well with the spectral color map shown in Figure 2b.

Comparison of Experimental Data with Non-Tilt Model Calculation

Figure S2 shows an overlay of the extracted LL transition energies (symbols from Figure 2b of the main text) with the calculated magneto-absorption spectra using the non-tilt model (Figure 3a). The observed transitions cannot be explained by the non-tilt model, particularly at low energies.

References

(1) Jiang, Y.; Dun, Z.; Moon, S.; Zhou, H.; Koshino, M.; Smirnov, D.; Jiang, Z. Landau quantization in coupled Weyl points: A case study of semimetal NbP. Nano Letters **2018**, 18, 7726–7731.

(2) Zhang, L.; Jiang, Y.; Smirnov, D.; Jiang, Z. Landau quantization in tilted Weyl semimetals with broken symmetry. Journal of Applied Physics **2021**, 129, 105107.

(3) Koshino, M. Cyclotron resonance of figure-of-eight orbits in a type-II Weyl semimetal. Physical Review B **2016**, 94, 035202.

(4) Jiang, Y.; Thapa, S.; Sanders, G. D.; Stanton, C. J.; Zhang, Q.; Kono, J.; Lou, W. K.; Chang, K.; Hawkins, S. D.; Klem, J. F.; Pan, W.; Smirnov, D.; Jiang, Z. Probing the semiconductor to semimetal transition in InAs/GaSb double quantum wells by magneto-infrared spectroscopy. Phys. Rev. B **2017**, 95, 045116.

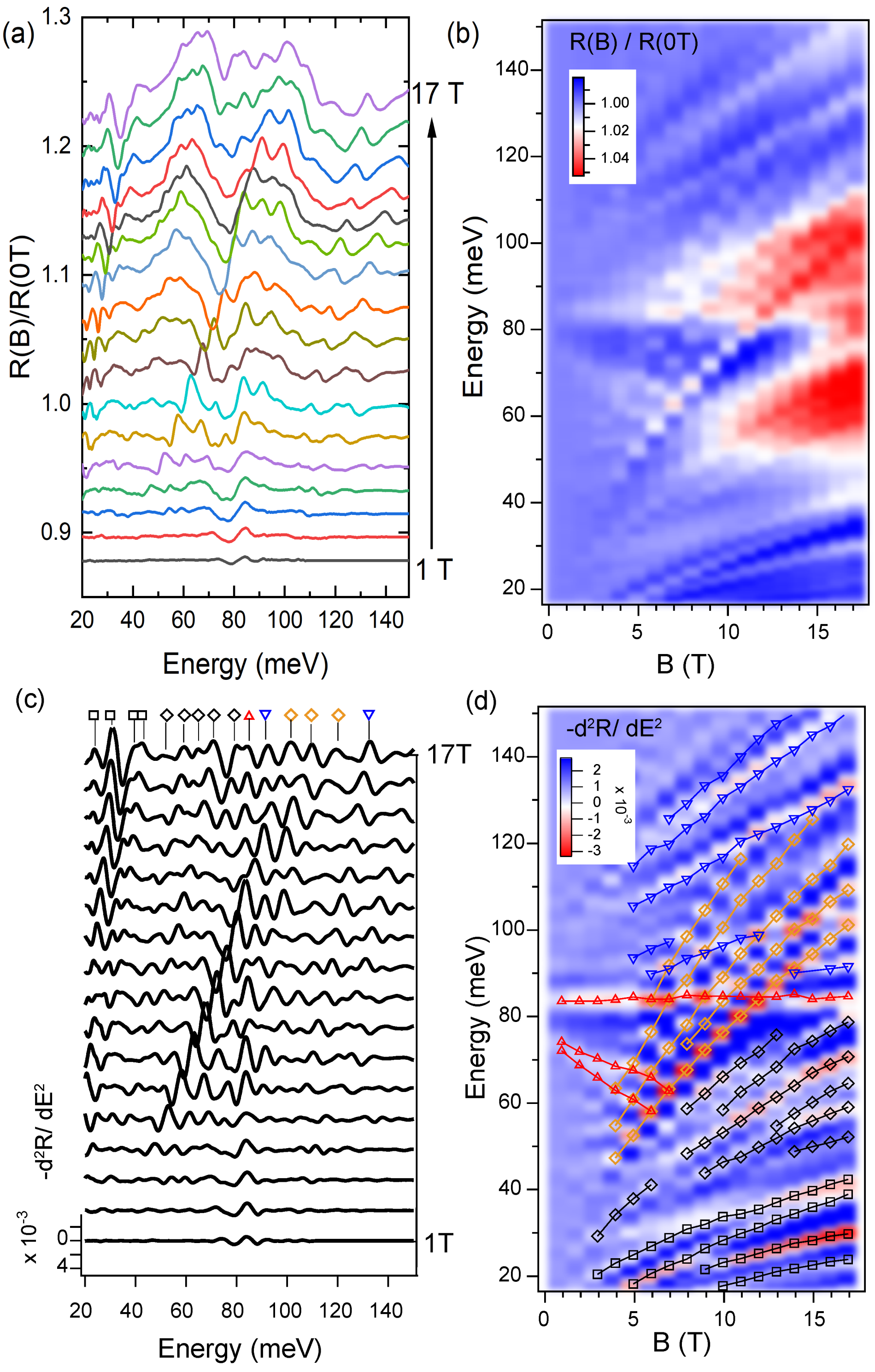

Figure S1: (a) Raw spectra of the magneto-reflectance measurements. The spectra are measured from 1 T to 17 T with a 1 T step increment, and they are offset for clarity. (b) Replot of Figure 2a in the main text. (c) Second derivative spectrum, $-d^2R/dE^2$, at each magnetic field. The spectra are offset for clarity, and the LL transitions are labeled by symbols above the 17 T spectrum. (d) Replot of Figure 2b in the main text.

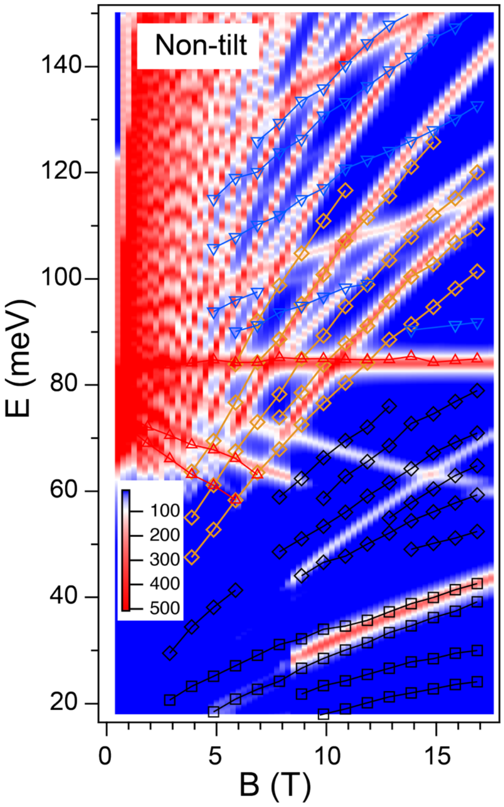

Figure S2: Overlay of the extracted LL transition energies (markers) on the calculated magneto-absorption spectra based on the non-tilt model.
